# Gender Differences in Bacterial STIs in Canada

**DOI:** 10.1186/1472-6874-4-S1-S26

**Published:** 2004-08-25

**Authors:** Thomas Wong, Ameeta Singh, Janice Mann, Lisa Hansen, Sharon McMahon

**Affiliations:** 1 Centre for Infectious Disease Prevention and Control, Health Canada, 400 Cooper Street, Suite 2005, Ottawa, Canada; 2 Infections Diseases Medical Consultant STD, Alberta Health and Wellness, 23rd Floor, Telus Plaza North Tower, Edmonton, Canada; 3 Centre for Infectious Disease Prevention and Control, Health Canada, Tunney's Pasture, Ottawa, Canada; 4 Centre for Infectious Disease Prevention and Control, Health Canada, Tunney's Pasture, Ottawa, Canada; 5 Centre for Infectious Disease Prevention and Control, Health Canada, Tunney's Pasture, Ottawa, Canada

## Abstract

**Health Issue:**

The incidence of bacterial sexually transmitted infections (STIs) is rising in Canada. If these curable infections were prevented and treated, serious long-term sequelae including infertility, and associated treatment costs, could be dramatically reduced. STIs pose a greater risk to women than men in many ways, and further gender differences exist in screening and diagnosis.

**Key Findings:**

Reported incidence rates of chlamydia, gonorrhea, and infectious syphilis declined until 1997, when the trend began to reverse. The reported rate of chlamydia is much higher among women than men, whereas the reverse is true for gonorrhea and infectious syphilis. Increases in high-risk sexual behaviour among men who have sex with men were observed after the introduction of potent HIV suppressive therapy in 1996, but behavioural changes in women await further research.

**Data Gaps and Recommendations:**

STI surveillance in Canada needs improvement. Reported rates underestimate the true incidence. Gender-specific behavioural changes must be monitored to enhance responsiveness to groups at highest risk, and more research is needed on effective strategies to promote safer sexual practices. Geographic and ethnic disparities, gaps, and needs must be addressed. Urine screening for chlamydia should be more widely available for women as well as men, particularly among high-risk men in order to prevent re-infections in their partners. As women are more likely to present for health examinations (e.g. Pap tests), these screening opportunities must be utilized. Female-controlled methods of STI prevention, such as safer topical microbicides, are urgently needed.

## Background

The incidence of sexually transmitted infections (STIs), such as chlamydia, gonorrhea and syphilis, is rising in Canada, with serious health and economic consequences. Women are at greater risk of STIs than men in a number of ways, including their susceptibility to infection and the severity of the sequelae associated with infection. A further difference between men and women lies in the difficulties involved in screening and diagnosis. This chapter discusses these differences and presents surveillance data on the incidence of chlamydia, gonorrhea and syphilis.

### STI Susceptibility and Transmission

A female in early adolescence is at increased risk of STIs, because her cervical os still comprises an exposed squamocolumnar junction, which during puberty is gradually replaced by squamous tissue[1]. Squamous cells are resistant to chlamydia and gonorrhea, whereas columnar epithelium supports their growth. As well, in early adolescence the cervical mucus, which is normally protective, is more easily penetrated by these organisms[2]. Oral contraceptive pills (OCPs) increase vulnerability to STIs in females of any age by enlarging the size of this squamocolumnar junction [3-6]. but may. protect against pelvic inflammatory disease (PID), a serious consequence of STIs [6-9].

Little is known about biological susceptibility factors in males, except that circumcision reduces the risk of STIs, including infection with human papillomavirus (HPV) [10-19]. Circumcision in men at high risk of HPV also reduces the risk of cervical cancer in their female partners[20].

Many STIs are transmitted more efficiently from males to females. For example, the risk of genital herpes transmission from a male to female partner is 19%, whereas it is 5% for transmission from female to male[21]. After a single episode of sexual intercourse, a woman has a 60% to 90% chance of contracting gonorrhea from her infected male partner, whereas the risk for a man from a woman is 20% to 30%[22,23]. The reasons for this difference may include greater exposure in females as a result of pooled semen in the vagina and greater trauma to tissues during intercourse.

### Sequelae

Once infected, women face a disproportionate burden of sequelae from STIs. These include PID, chronic pelvic pain, ectopic pregnancy, infertility and cervical cancers. After one episode of PID, 20% of females will suffer chronic pelvic pain, 9% an ectopic pregnancy and 8% infertility; the risk of infertility doubles after each subsequent episode[24]. PID is the cause of 15% of all infertility. In men, infertility is only a very rare complication of STIs. Although HPV rarely causes penile and anal cancers in males, females may develop the much more common cervical as well as vaginal and anal cancers[25]. If a pregnant woman is infected, the STI can be transmitted to her newborn, and may result in miscarriage, premature labour, stillbirth, mental retardation and infant death[26].

### Screening, Diagnosis and Treatment

For gonorrhea, up to 80% of females can be asymptomatic, as compared with about 10% to 20% of men[27]. Thus, females are more likely than males to harbour this infection, and it is likely to remain undiagnosed. Although 80% of women with chlamydia remain asymptomatic, half of men with chlamydia have symptoms[28,29]. Screening is critical to reducing the size of this hidden epidemic. Indeed, a randomized controlled trial of chlamydia screening demonstrated a reduction in the incidence of PID[30].

Clinical diagnosis of STIs in females is not as reliable as in males because invasive pelvic examinations are required and because vaginal discharge may be due to non-sexually transmitted diseases. As well, laboratory detection of an STI in genital specimens from women is more difficult: the sensitivity and specificity of the tests used may be affected by the presence of blood, mucus or organisms normally present in the body[31].

Males may be less likely to attend for screening, as collecting a urethral swab might be a deterrent. STI recurrences in females have been associated with an infected male partner who either keeps the infection secret or whose condition remains undiagnosed and untreated. Chlamydia screening programs have traditionally targeted only females. Ironically, if active screening of the infection in men were increased this would benefit women by reducing their reinfection rate and sending the message that men must take responsibility for sexual and reproductive matters. The recent introduction of non-invasive urine-based nucleic acid diagnostic tests, which may be carried out in or outside the health care setting, has overcome certain diagnostic challenges in both males and females [32-38], and these tests may prove less of a deterrent to screening in men.

As settings of both prevention and treatment, STD clinics have been less sensitive to the needs of women, whereas family planning programs have focused less on the needs of men[39]. Men are more likely than women to be screened for syphilis when an STI is suspected[40]. Women are more likely to treat themselves, using over-the-counter vaginal yeast medications, douches, etc., which can mask an actual STI and delay diagnosis[39,41].

## Methods

Chlamydia, gonorrhea and syphilis infections are nationally notifiable bacterial STIs in Canada. All 13 provinces and territories provide Health Canada with non-nominal surveillance data consisting of at least age or age group, sex, and province or territory of residence.

Surveillance data submitted to Health Canada from 1991 to 2000 (From 1993 infectious syphilis (primary, secondary and early latent syphilis) was introduced to replace early symptomatic syphilis; chlamydia data were reported only from 1992 by some jurisdictions were analyzed. The data for 2000 are preliminary, and changes for this year are anticipated because of reporting delays. Annual incidence rates per 100,000 population were calculated using census population estimates and intercensus estimates from Statistics Canada. The chi-square test was used for comparison of proportions, and the chi-square test for trends was used to compare proportion trends. A two-sided *p *value of less than 0.05 was considered to indicate statistical significance.

## Results

### Chlamydia (Figures 1 and 2)

**Figure 1:**
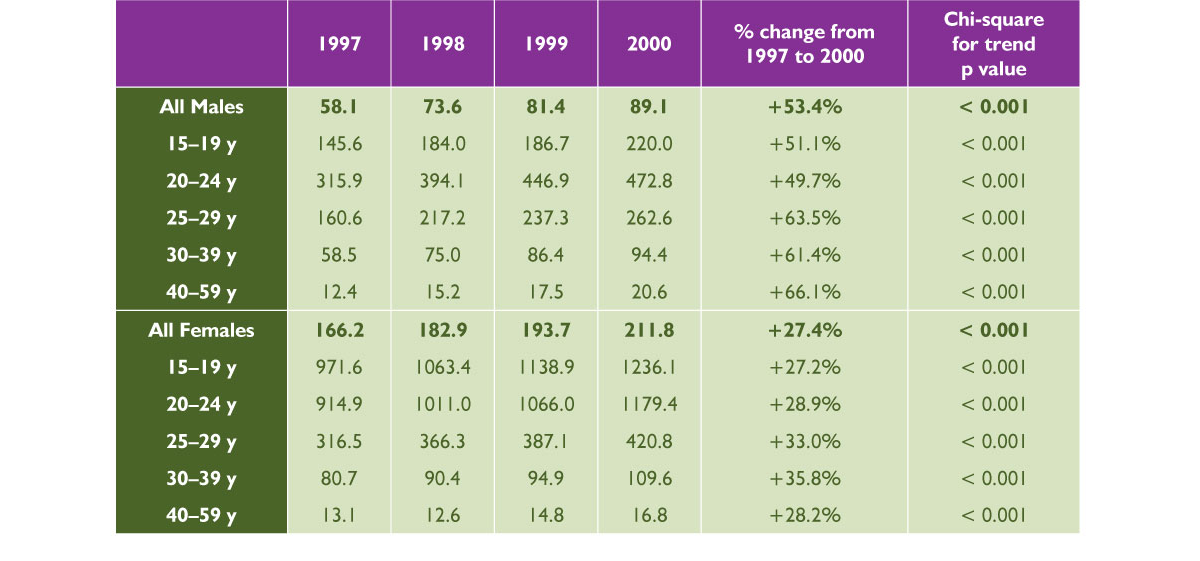
**Reported Chlamydia Rate per 100,000 Population in Canada, 1997–2000 **Source: Sexual Health and Sexually Transmitted Infections, Centre for Infectious Disease Prevention and Control, Health Canada, 2001

**Figure 2:**
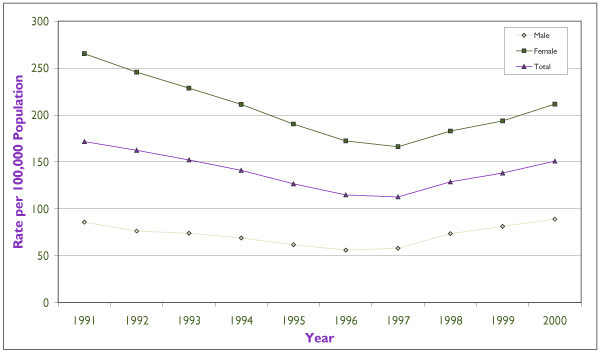
**Reported Chlamydia Rates in Canada **Source: Sexual Health and Sexually Transmitted Infections, Centre for Infectious Disease Prevention and Control, Health Canada, 2001

Females accounted for over two thirds of the 46,000 chlamydia cases reported in Canada, at a rate of 211.8 per 100,000 versus 89.1 per 100,000 among men in the year 2000 (*p *< 0.001). The chlamydia rate had been on the decline until 1997, when this downward trend started to revert (Figure 1). Since then, the rate has increased significantly among both males (*p *< 0.001) and females (*p *< 0.001), with a greater increase in males (53.4% versus 27.4%, *p *<0.001). The highest incidence and fastest rate of increase has been in the 15 to 24 age group among females but in the 20s age group among males.

### Gonorrhea (Figures 3 and 4)

**Figure 3:**
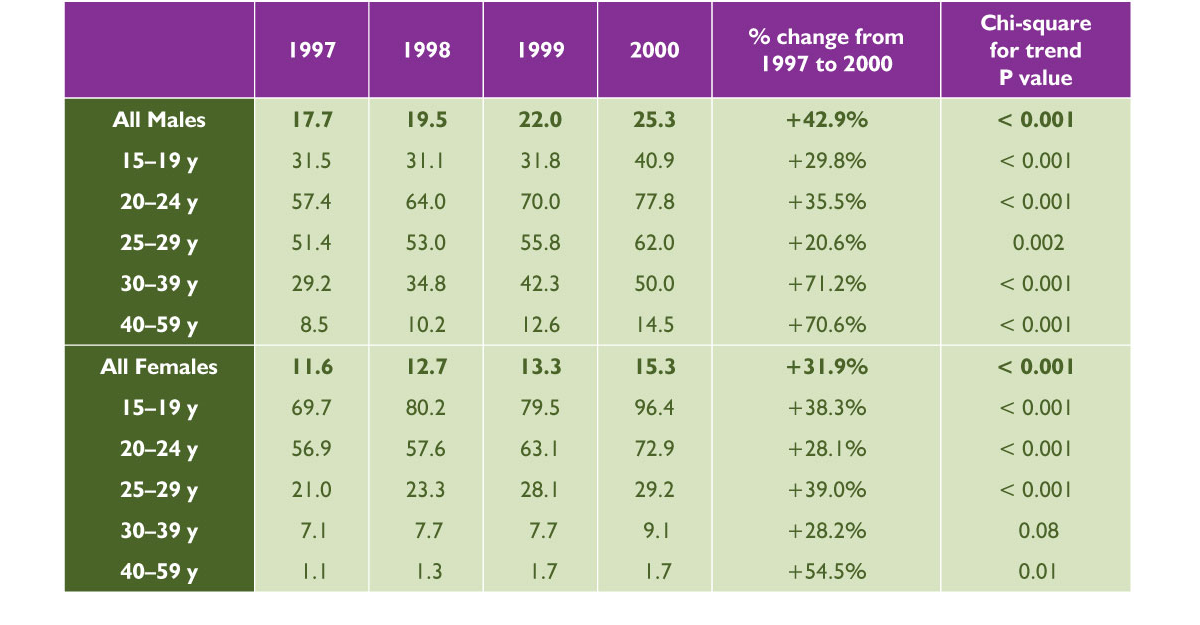
**Reported Gonorrhea Rate per 100,000 Population in Canada, 1997 to 2000. **Source: Sexual Health and Sexually Transmitted Infections, Centre for Infectious Disease Prevention and Control, Health Canada, 2001

**Figure 4:**
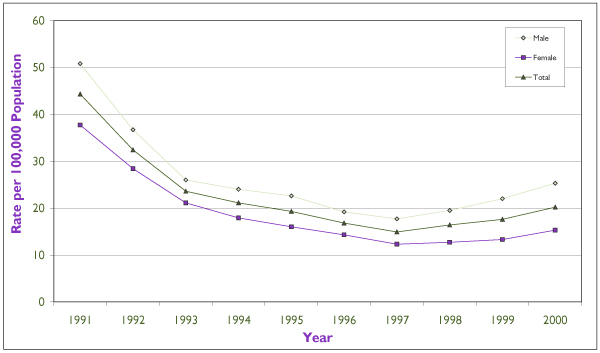
**Reported Gonorrhea Rates in Canada **Source: Sexual Health and Sexually Transmitted Infections, Centre for Infectious Disease Prevention and Control, Health Canada, 2001

Males accounted for almost two thirds of the 6,000 nationally reported cases of gonorrhea in the year 2000, at a rate of 25.3 per 100,000 versus 15.3 per 100,000 among females (*p *< 0.001). The gonorrhea rate had been on the decline until 1997, when it began to increase again (Figure 2). Since then, the rate has increased significantly among both males (*p *< 0.001) and females (*p *= 0.02), but with a greater increase among males (42.9% versus 31.9%, *p *= 0.002). Among females, the peak incidence and the fastest rate of increase have taken place in the 15 to 24 age group. The highest reported incidence among males has been in the 20 to 29 age group and the fastest rate of increase in the 20s and 30s age groups.

### Syphilis (Figures 5 and 6)

**Figure 5:**
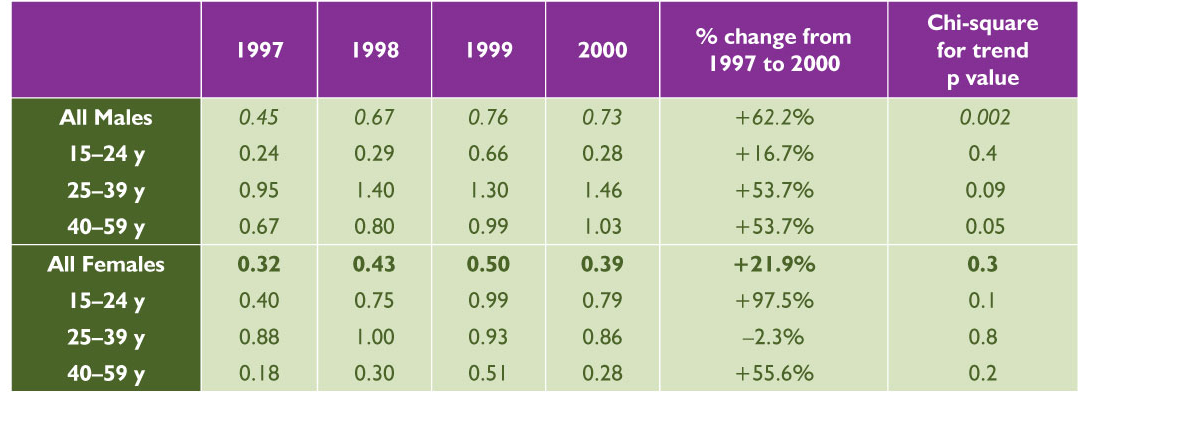
**Reported Infectious Syphilis Rate per 100,000 Population in Canada, 1997 to 2000 **Source: Sexual Health and Sexually Transmitted Infections, Centre for Infectious Disease Prevention and Control, Health Canada, 2001

**Figure 6:**
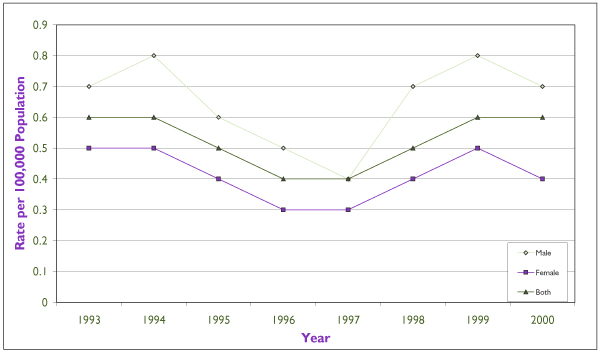
**Reported Infectious Syphlis Rates in Canada **Source: Sexual Health and Sexually Transmitted Infections, Centre for Infectious Disease Prevention and Control, Health Canada, 2001

Males made up almost two thirds of the 171 cases of infectious syphilis reported nationally in the year 2000, at a rate of 0.73 per 100,000 versus 0.39 per 100,000 among females (*p *< 0.001). The infectious syphilis rate had been on the decline until 1997, when this downward trend started to revert (Figure 3). Since then, the rate has increased significantly among males, by 62.2% (*p *< 0.001), but not among females (*p *= 0.3). The peak incidence among females occurred in 15 to 39 year olds and the fastest rate of increase in the 15 to 24 age group. The highest incidence and fastest increase among males have occurred in the 25 to 29 age group.

## Discussion

### STI

Since 1996, molecular diagnostic testing for chlamydia has become increasingly available across Canada. These tests have the advantage of being non-invasive and more sensitive than the older methodologies used for the diagnosis of chlamydia. In addition, the increasing availability of urine-based tests has made testing for chlamydia more acceptable for both men and women. This, along with recent improvements in chlamydia screening initiatives, may have contributed to the observed increase in reported chlamydia incidence.

Since the introduction of these tests, there has been a consistent upswing in reported rates not only of chlamydia but also of gonorrhea and syphilis, despite the lack of widespread screening and diagnostic advances for these latter infections in Canada. In the absence of improvement in disease reporting, this is strongly suggestive of real increases in the occurrence of these STIs in recent years.

### Sexual Behaviours

Although STI rates had been declining since the beginning of the HIV epidemic – possibly as a result of prevention and screening programs, changes in sexual behaviour in response to the HIV epidemic, and the availability of single-dose treatment for some STIs[42,43] – the decline. was reversed in 1997. Increases in high-risk sexual behaviour in men who have sex with men were reported after the introduction of potent, antiretroviral HIV suppressive therapy [44-47], but behavioural changes in women await further research.

Girls and boys are socialized into different gender roles, affected by culture, peer and parental influences. Males tend to have an earlier sexual debut and a higher rate of partner changes[39,48]. Females are more likely than males to have been forced into their first sexual encounter[49]. Men have more control than women over condom use. Women are uncomfortable about insisting on condom use for fear of the reactions of their male partners regarding trust and commitment issues[50,51]. The power differential and potential for domestic violence create barriers that may prevent women from protecting themselves[52]. Therefore, prevention programs for women need to target safer sex negotiation skills and female-controlled methods[53,54].

### Prevention

In a U.S. survey of 1,000 females, only 11% were aware of the fact that STIs are more harmful to females than to males; those with less knowledge were less likely to practise safer sex[39]. Preventive information is necessary for behaviour to change, but it is not sufficient. Most prevention programs have been proven to fail. Issues specific to gender and culture must be incorporated. Women benefit from prevention strategies that address gender roles, the power differential between the sexes, financial dependence on men, fulfillment in their intimate relationships, and bonding with other women [55-57]. Two randomized controlled trials incorporating these elements showed a significant reduction in rates of STIs[58,59].

### Economic Burden

In 1990, the estimated direct and indirect costs of chlamydia in Canadian females, including diagnosis, treatment and productivity loss, were as high as $115 million (Canadian) dollars; for males the cost was as high as $8 million. In the same year, the cost burden of gonorrhea was up to $63 million for females and $12 million for males[60]. If properly prevented and treated, the cost savings would be enormous for these easily curable illnesses.

### Data Limitations

The reported rate for any disease may underestimate the true incidence if a lack of symptoms causes people not to present themselves for diagnosis. Up to 80% of women with gonorrhea or chlamydia can be asymptomatic, and discharge and genital lesions are harder to detect and diagnose in women. In general, men do not go for screening or medical examination as regularly as women and, even if they do, clinicians may not screen them for STIs if they are asymptomatic. Therefore, undiagnosed chlamydia in men may contribute in part to the significantly lower reported rate among men. Thus the reported rates are a fraction of the true disease burden, and they are influenced by the amount of screening.

### Policy Implications and Recommendations

The highest public health priority is to reduce this hidden epidemic in youth, the group burdened with the highest infection rate. Young women are more susceptible to STIs than young men, and more likely to have hidden infections, resulting in longer diagnostic and treatment delays and the development of long-term sequelae. Health professionals should be encouraged to provide prevention and care services that are evidence-based, gender-specific and culturally sensitive. To reduce the health and economic consequences of the increasing rates of STIs, action must be taken in the following areas.

#### Surveillance

• Strengthen the STI surveillance system across Canada, especially in high-risk subpopulations.

• Assess gender, ethnic (e.g. Aboriginal people) and geographic disparities (e.g. rural versus urban).

#### Research

• Evaluate gender-specific behavioural changes leading to increased STI rates.

• Evaluate safer, acceptable and female-controlled methods of STI prevention, such as topical microbicides, that do not increase the risk of HIV transmission[61].

• Evaluate gender-specific strategies that promote safer sexual practices and behaviour change, such as the use of the Internet for STI/HIV "cyberprevention."

#### Prevention

• Develop gender-specific strategies that address biological, socio-demographic, access, prevention, screening and behavioural issues[62].

• Increase availability of urine screening for chlamydia.

• Improve urine screening test performance for gonorrhea.

• Increase prevention efforts targeting the high disease burden of chlamydia, particularly through screening of youth and disenfranchised populations as well as of women and men at high risk.

• Increase opportunities for screening by taking advantage of women's greater tendency to present for health examinations (e.g. Pap tests).

• Adopt creative approaches to screening in men, in order to prevent reinfection in their female partners.

## Note

The views expressed in this report do not necessarily represent the views of the Canadian Population Health Initiative, the Canadian Institute for Health Information or Health Canada.
